# Heterogeneous induction of microglia M2a phenotype by central administration of interleukin-4

**DOI:** 10.1186/s12974-014-0211-6

**Published:** 2014-12-31

**Authors:** Giovanna Pepe, Giorgia Calderazzi, Marcella De Maglie, Alessandro Maria Villa, Elisabetta Vegeto

**Affiliations:** Center of Excellence on Neurodegenerative Diseases, Department of Pharmacological and Biomolecular Sciences, University of Milan, via Balzaretti, 9, 20133 Milan, Italy; Department of Veterinary Science and Public Health Veterinary Medicine, University of Milan, Via Celoria, 20133 Milan, Italy

**Keywords:** alternative activation, centrally administered interleukin-4, M2 polarization, microglia

## Abstract

**Background:**

Acquisition of the M1 or M2 phenotypes by microglia has been shown to occur during the development of pathological conditions, with M1 activation being widely involved in neurotoxicity in relation with the anatomical localization and the reactivity of subtypes of microglia cells. On the contrary, little is known on the ability of microglia to undergo M2 polarization by interleukin-4 (IL4), the typical M2a polarization signal for peripheral macrophages.

**Methods:**

Recombinant mouse IL4 was injected in the third cerebral ventricle of mice to induce brain alternative polarization. The mRNA levels of *Fizz1*, *Arg1*, and *Ym1* genes, known to be up-regulated by IL4 in peripheral macrophages, together with additional polarization markers, were evaluated in the striatum and frontal cortex at different time intervals after central administration of IL4; in parallel, M2a protein expression was evaluated in tissue extracts and at the cellular level.

**Results:**

Our results show that the potency and temporal profile of IL4-mediated M2a gene induction vary depending on the gene analyzed and according to the specific brain area analyzed, with the striatum showing a reduced M2a response compared with the frontal cortex, as further substantiated by assays of polarization protein levels. Of notice, *Fizz1* mRNA induction reached 100-fold level, underscoring the potency of this specific IL4 signaling pathway in the brain. In addition, immunochemistry assays demonstrated the localization of the M2 response specifically to microglia cells and, more interestingly, the existence of a subpopulation of microglia cells amenable to undergoing M2a polarization in the healthy mouse brain.

**Conclusions:**

These results show that the responsiveness of brain macrophages to centrally administered IL4 may vary depending on the gene and brain area analyzed, and that M2a polarization can be ascribed to a subpopulation of IL4-responsive microglia cells. The biochemical pathways that enable microglia to undergo M2a activation represent key aspects for understanding the physiopathology of neuroinflammation and for developing novel therapeutic and diagnostic agents.

**Electronic supplementary material:**

The online version of this article (doi:10.1186/s12974-014-0211-6) contains supplementary material, which is available to authorized users.

## Background

Microglia are myeloid cells that populate the parenchyma of the central nervous system (CNS); their physiological activity includes most of the biological properties that are typical for peripheral macrophages, although their developmental origin and anatomical distribution allows these cells to perform distinctive immune and neuromodulatory functions in the CNS. Through their physical and biochemical interaction with neurons, microglia are able to sense and remodel neuronal activity, support neurogenesis, and maintain CNS homeostasis [1].

Microglia also possess the striking ability to rapidly react to endogenous or exogenous signals with a variety of physiological responses [2]. Like peripheral macrophages, microglia are activated by bacterial or viral signals to acquire a classical ‘M1’ reactive phenotype that defeats invading pathogens through the activation of a wide range of reactions, such as the release of reactive oxidizing species and inflammatory mediators; a large body of evidence shows that the chronic or unrestrained M1 activation of microglia results in neurotoxicity, as thoroughly demonstrated by the use of the bacterial endotoxin lipopolysaccharide in several experimental settings and *in vivo* [3-6]. The neurotoxic potential of microglia M1 activation may vary depending on region-specific signals, as in the case of dopaminergic neurons of the nigrostriatal pathway, which strongly influence the outcome of neuroinflammation through pathogenic mechanisms that include dopamine metabolism and oxidative stress [7,8]. Furthermore, the inflammatory M1 phenotype, similarly to resting activities, has been recently ascribed to subtypes of microglia that, residing adjacent to each other within the same brain region, take on certain tasks and not others [9]. This functional heterogeneity among microglia has, so far, been documented by cell distribution and morphology [10], neural-immune communication [11-14], and response to neurotransmitters [15] and lipopolysaccharide [16,17].

Conversely, macrophages and microglia undergo an alternative ‘M2’ phenotype as a consequence of parasite invasion and in response to the endogenous immune signals, interleukin-4 (IL4) and interleukin-13 (IL13), in order to provide tissue repair and resolution of inflammation. A more detailed analysis led to the identification of two distinct alternative activation states, M2a and M2c, which reflect the actions of IL4 and IL13, on the one hand, and interleukin-10 (IL10) and TGFβ, on the other hand; importantly, these responses are associated with specific panels of regulated genes and a distinct range of effects [18]. However, only a few reports have addressed the study of M2 polarization in CNS physiopathology and current knowledge mainly derives from *in vitro* or *ex vivo* studies, showing the responsiveness of microglia to IL4 and the subsequent activation of gene expression programs related to neuroprotection, tissue remodeling, and angiogenesis [19]. As a consequence, it is not known whether microglia are able to sustain alternative activation equally throughout the CNS or whether heterogeneous subtypes of M2 responder microglia exist within the same anatomical location. Considering the contribution of the M2 phenotype to neuroprotection, it is indeed mandatory to fill this gap of knowledge and reach a wider view of microglia reactiveness and its involvement in the pathogenesis of neurologic diseases, particularly those that show a region-specific pattern of development together with microglia activation, such as Parkinson’s disease [20]. Furthermore, the study of microglia M2 phenotype will provide biochemical details for the identification of novel pharmacological and diagnostic tools that target microglia activation processes [9].

The aim of this study was thus to investigate microglia M2 activation *in vivo* and evaluate the responsiveness and functional heterogeneity of microglia to undergo M2 polarization among and within selected brain areas. We thus stimulated microglia by central administration of IL4 in the third cerebral ventricle of the mouse brain. Interestingly, our data show that the induction of microglial M2a phenotype varies among the two brain areas analyzed, with M2a gene expression being more potent in the frontal cortex than in the striatum, and that only a subpopulation of microglia cells within these areas is amenable to undergoing an M2a response. Finally, we show that, among the M2a genes tested, *Fizz1* reached the highest induction levels, underscoring the potency of this IL4 signaling pathway in the brain.

## Methods

### Materials

Recombinant mouse IL4 was obtained from PeproTech (London, UK). Unless otherwise specified, chemicals were purchased from Merck (Darmstadt, Germany).

### Animals

C57BL/6− male mice 4 months of age were supplied by Charles River Laboratories (Calco, Italy). All animals were allowed free access to food and water and were kept in temperature-controlled facilities on a 12-hour light and dark cycle. Animals were housed in the animal care facility of the Department of Pharmacological and Biomolecular Sciences at the University of Milan, and experiments were performed in compliance with regulations approved by the Institutional Animal Care and Use Committee of the University of Milan and in accordance with European legislation.

### Preparation of bone-marrow-derived macrophages

Bone-marrow-derived macrophages were prepared as previously described [21]. Briefly, bone marrow from the tibia and femur was flushed with RPMI (Life Technology; Monza, Italy) using a 21 gage needle. Cells were centrifuged at 1200 rpm for 5 min at 10°C, seeded in tissue culture plates, and grown for 7 days in DMEM containing 20% endotoxin-free FBS, 30% L929-cell conditioned media, 1% L-glutamine, 1% penicillin and streptomycin, and 0.5% Na pyruvate. On the day of the experiment, cells were treated for 24 h with 20 ng/ml of IL4 and RNA prepared as described.

### Intracerebroventricular injections

Intracerebroventricular (icv) injections were made as previously described [17]. Briefly, mice were deeply anesthetized with a subcutaneous injection of a mixture of ketamine and xylazine (78 and 6 mg/kg, respectively) and positioned on a specific stand for the surgical operation. Injections in the third cerebral ventricle (icv) were performed according to specific stereotaxic coordinates (bregma, −0.25 mm; lateral, 1 mm; depth, 2.25 mm), as previously described [17]. Interleukin-4 was injected in 2.5 μl of 0.9% NaCl using a 26S-gage Hamilton Syringe; 100 and 250 ng were injected to assess RNA and protein levels, respectively. Infusions were made at a rate of 0.1 μl in 3 s. The needle was kept in place for 30 s after the injection and then removed slowly. Animals injected with the same volume of vehicle (0.9% NaCl) alone were used as controls. The skin incision was closed with a suture and animals were allowed to recover for 8, 16, 30 or 48 hours before sacrifice by a lethal ketamine and xylazine solution (150 and 12 mg/kg, respectively). For RNA quantification, the right striatum and frontal cortex, contralateral to the injection site, were collected, immediately frozen on dry ice, and stored at −80°C until RNA preparation. For immunological assays, the right hemisphere was processed for immunohistochemistry, while ipsilateral areas were frozen on dry ice and stored at −80°C for Western blot analysis.

### RNA and cDNA preparation

The striatum and frontal cortex were homogenized using steel beads and tissue Lyser (QIAGEN, Milan, Italy) at 28 Hz, for three cycles of 20 s followed by 30 s, on ice and in RLT buffer and total RNA was purified using RNeasy minikit (QIAGEN) according to the manufacturer’s instructions including a step with deoxyribonuclease incubation. One μg RNA was used for cDNA preparation using 8 U/μl of Moloney murine leukemia virus reverse transcriptase (Promega, Milan, Italy) in a final volume of 25 μl; the reaction was performed at 37°C for 1 h, and the enzyme inactivated at 75°C for 5 min. Control reactions without the addition of the reverse transcription enzyme were performed (data not shown).

### Real time PCR

A 1:16 cDNA dilution was amplified using SYBR technology. The PCR was carried out in triplicate or duplicate on a 96-well plate using GoTaq®qPCR Master Mix technology (Promega) according to the manufacturer’s protocol using 7900HT fast real time PCR system (Applied Biosystems, Life Technologies) with the following thermal profile: 2 min at 95°C; 40 cycles, 15 s at 95°C, 1 min at 60°C. Gene expression of target genes was assessed for *arginase-1* (*arg1*; forward primer, 5′-CAGAAGAATGGAAGAGTCAG-3′; reverse primer, 5′-CAGATATGCAGGGAGTCACC-3′), *chitinase-like 3* (*Chi3l3*, or *Ym1*; forward primer, 5′-GAAGGAGCCACTGAGGTCTG-3′; reverse primer, 5′-GAGCCACTGAGCCTTCAAC-3′), *Found-in-the inflammatory zone* (*Fizz1*, or *Retnlα*; forward primer, 5′-GGAACTTCTTGCCAATCCAGC-3′; reverse primer, 5′-AAGCCACAAGCACACCCAGT-3′), *IL4 receptor-α* (*IL4Rα*; forward primer, 5′-AACTCGCAGGTTCTGGCTGG-3′; reverse primer, 5′-AAGCCCCGAGTCCTAGGTT-3′), *CD206* (forward primer, 5′-TTCAGCTATTGGACGCGAGG-3′; reverse primer, 5′-GAATCTGACACCCAGCGGAA-3′), *TGFβ* (forward primer, 5′-ACCAACTATTGCTTCAGCTTCAGCTCCAC-3′; reverse primer, 5′-GATCCACTTCCAACCCAGGTC-3'), *IL1β* (forward primer, 5′-TGCCACCTTTTGACAGTGATG-3′; reverse primer, 5′-GCTGCGAGATTTGAAGCTGG-3′), *TNFα* (forward primer, 5′-CCTATGTCTCAGCCTCTTCTC-3′; reverse primer 5′-CTCTTGCTTATCCCCTCTTCC-3′), and for the reference genes *36B4* (forward primer, 5′-GGCGACCTGGAAGTCCAACT-3′; reverse primer, 5′-CCATCAGCACCACGGCCTTC-3′) and *complement component 1qA* (*C1qA*; forward primer, 5′-GACCACGGAGGCAGGGACAC-3′; reverse primer 5′-CTTCCCGTTGGGTGCTCGGC-3′). The reactions were carried out according to the manufacturer's protocol using a 7900HT fast real time PCR system (Applied Biosystems, Inc.), and the data were analyzed using the 2^-ΔΔCt^ method.

### Western blotting

Brain tissues were homogenized using steel beads with a tissue Lyser (QIAGEN) at 28 Hz, for 3 cycles of 20 s followed by 30 s, on ice and in a buffer for total cellular extracts, containing 5 mM MgCl_2_, 20 mM HEPES (pH 7.9), 420 mM NaCl, 0.1 mM EDTA, 20% glycerol, 0.1% Triton, 5 mM β-mercaptoethanol, 0.1 mM PMSF, 10 μg/ml aprotinin, and 1 μg/ml leupeptin. The lysates were frozen on dry ice for 5 min and then thawed at 37°C for 5 min for three times. The samples were centrifuged at 13,000 rpm at 4°C for 20 min and the supernatants were collected and stored at −20°C. Protein concentration was estimated by Bradford protein assay using BSA as standard. Equal amounts of protein (20 μg) were dissolved in Laemmli’s sample buffer, boiled for 5 min, and separated with a SDS-polyacrylamide minigel (10% and 7.5% polyacrylamide for Ym1 and CD206 detection, respectively) and then transferred overnight at 15 mA into 0.45 μm Hybond-ECL membrane (GE Healthcare, Milan, Italy). Membranes were incubated for 1 h with blocking solution containing 5% (w/v) nonfat milk in Tris-buffered saline (TBS) and subsequently probed for 1 h at room temperature with a rabbit anti-mouse YM1 antibody (1:1000; Stem Cell Technologies, Grenoble, France) in incubation solution (TBS containing 5% (w/v) nonfat milk and 0.1% Tween 20. After extensive washing in TBST (TBS + 0.1% Tween 20), or goat anti-mouse MMR/CD206 polyclonal antibody (CD206 (1:500, R&D Systems, Minneapolis, MN, USA). Blots were incubated with horseradish peroxidase-conjugated goat anti-rabbit IgG (1:2000, for Ym1 detection) or horse anti-goat IgG (1:2000, for CD206 detection; both from Vector Laboratories, Peterborough, UK) in incubation solution, for 1 h at room temperature. After extensive washing in TBST, immunoreactive bands were visualized using a chemiluminescence assay detection system according to the manufacturer’s instructions (Amersham™ ECL™ Western Blotting Analysis System, GE Healthcare). To ascertain that blots were loaded with equal amounts of protein lysates, they were also incubated in the presence of the antibody against β-actin protein (1:10,000; Sigma-Aldrich Corp., Milan, Italy). Subsequently, for semiquantitative analyses, the densities of the protein bands of YM1 (45 kDa), CD206 (175 kDa), and β-actin (42 kDa) were measured by densitometric scanning of the membrane with Gel Doc™ XR Imaging Densitometer (Bio-Rad Lab, Segrate, Italy) and a computer program (Quantity One® software, Bio-Rad Lab). Western blotting images were arranged in the final figures using Microsoft software.

### Immunohistochemistry

All immunohistochemical analyses were performed on animals treated for 16 h with IL4. Right brain hemispheres were removed and fixed overnight at 4°C by immersion in 4% formalin solution.

#### Cryoprotection

Brains were immersed at 4°C in 30% sucrose solution until they sank, embedded in optimal cutting temperature compound, and stored at −80°C until analysis. Coronal sections of brain 20 μm thick were collected using a cryostat (Microm HM 505E, Walldorf, Germany). Free-floating sections were washed five times with TBS + 0.01% Triton and incubated with blocking solution (TBS + 10% goat serum + 0.4% Triton) for 1 hour at room temperature. Next, sections were incubated overnight with the following antibodies, diluted in TBS with 1% goat serum: rabbit anti-mouse antibody against Ym1 (1:50 dilution, Stem Cell Technologies), rat anti-mouse antibody against macrophage antigen complex-1 Mac-1 (1:200; Serotec, Puccheim, Germany), mouse anti-mouse antibody against feminizing locus on X-3 (NeuN; 1:100; Merck), mouse anti-mouse glial fibrillary acidic protein (GFAP; 1:500; Sigma-Aldrich). Sections were washed five times with TBS + 0.01% Triton and incubated for 2 h with secondary antibodies (1:200 AlexaFlour 488 for Mac1, NeuN, and GFAP; 1:200 AlexaFlour 555 for Ym1; Molecular Probes, Monza, Italy) at room temperature. Sections were washed five times with TBS + 0.01% Triton and then incubated for 15 minutes with Hoechst stain (Sigma-Aldrich). In parallel, some sections were tested for antibody specificity by omitting primary or secondary antibodies. After five washings in TBS + 0.01% Triton, sections were mounted on slides and observed using a Zeiss Axioskop microscope equipped with a digital camera (Carl Zeiss, Thornwood, NY); images were captured at 40,000× and 63,000× magnification. Quantification of Ym1-positive microglia cells was performed by counting the number of cells showing a red-labeled Ym1 signal and a green-Mac-1 signal; cells were scored as positive on the basis of nuclear DAPI staining in close proximity. Four counting fields of 50 × 50 μm were analyzed in two sections from three different levels, at least 120 μm apart, of the striatum and frontal cortex (*n* = 3).

#### Paraffin embedding

Brains were trimmed using a brain matrix (Adult Mouse Brain Slicer Matrix BSMAS005-1, Zivic Instruments, Pittsburgh, PA, USA) and sections were routinely processed, paraffin embedded, and sectioned in 4 μm serial sections. For Iba1 and Arg1 immunohistochemistry, sections were immunostained with rabbit polyclonal anti-Iba1 antibody (ionized calcium-binding adapter molecule-1, 019-19741, Wako Chemicals, Richmond, VA, USA), and goat polyclonal anti-Arg1 antibody (sc-18354, Santa Cruz Biotechnology, Heidelberg, Germany); sections were than incubated with biotinylated goat anti-rabbit (Iba1) and rabbit anti-goat (Arg1) secondary antibodies (VC-BA-1000-MM15 and VC-BA-5000-MM15, Vector Laboratories, Petersborough, UK), labeled by the avidin-biotin-peroxidase procedure with a commercial immunoperoxidase kit (VECTASTAIN® Elite ABC-Peroxidase Kit Standard, VC-PK-6100-KI01, Vector Laboratories). The immunoreaction was visualized with DAB (Peroxidase DAB Substrate Kit, VC-SK-4100-KI01, Vector Laboratories) substrate and sections were counterstained with Mayer’s hematoxylin (C0302, Diapath, Italy). Digital image analysis was performed by scoring the number of Iba1- and Arg1-positive cells (microglia) in three 400× microscopic fields in the frontal cortex of both vehicle and IL4 treated mice (*n* = 3).

### Microglia sorting and fluorescence-activated cell sorting analysis

After 16 h of IL4 or vehicle treatment, brains were dissected and washed in Hank’s Balanced Salt Solution (HBSS; Life Technologies); after removing the meninges, cortices from five mice were pooled as a single experimental group. Enzymatic cell dissociation was performed using Neural Tissue Dissociation Kit P (Miltenyi Biotec, Bologna, Italy), following a modified version of the protocol supplied by the manufacturer. Briefly, after enzymatic digestion with papain, samples were dissociated mechanically, homogenized, and filtered through a 40-μm cell strainer. After extensive washes in HBSS, myelin was removed by centrifuging the dissociated brain cells, which had previously been suspended in 10 ml of cold 0.9 M sucrose solution, at 850 *g* and 4°C for 10 min without braking. Floating myelin and the supernatant were discarded and cells were processed for microglia magnetic sorting by incubating with CD11b MicroBeads (diluted 1:10 in PBS + 0.05% BSA; Miltenyi Biotec) for 15 min at 4°C; after washings, cells were suspended in 500 μl of PBS + 0.05% BSA and applied to a magnetic column to purify CD11b^+^ cells, namely microglia. Immediately after isolation of microglia, cells were fixed with 4% paraformaldehyde, extensively washed with 125 mM glycine in PBS and permeabilized overnight in PBS containing 0,1% Triton X-100, 5% normal goat serum, and 2% BSA, at 4°C. Cells were incubated with rabbit anti-mouse Ym1 antibody (Stem Cell Technologies) diluted 1:50 in Incubation Solution (PBS containing 0.1% Triton X-100, 1% normal goat serum, and 2% BSA) at room temperature for 1 h. After extensive washes in incubation solution, cells were incubated with Alexa633-conjugated anti-rabbit secondary antibody (1:200 in incubation solution; Molecular Probes) for 1 h at room temperature. Cells were extensively washed with PBS and then analyzed with a flow cytometry system (BD FACSCalibur, Becton Dickinson Biosciences, San Jose, CA, USA). Isotype IgG controls were also used to evaluate nonspecific signals. Incubations with FITC-antiCD11b antibody (Miltenyi Biotec) and flow cytometry analyses were performed separately on dissociated brain cells as well as on CD11b-immunosorted cells to calculate recovery and purity; our protocol allowed us to obtain 80 to 85% recovery of CD11b-positive microglia cells, that is, 12% of the total brain cells population after tissue dissociation and myelin removal, with 90 to 95% purity after magnetic immunosorting.

### Statistical evaluation

Unless otherwise stated, all values are expressed as mean ± standard error of the mean (SEM) of *n* observations. The results were analyzed by one-way ANOVA followed by a Bonferroni post-hoc test for multiple comparisons using GraphPad Prism 5 software [22]. A value of *P* <0.05 was considered significant.

## Results

### Region-specific differences in IL4-induced M2 gene expression

**Figure 1:**
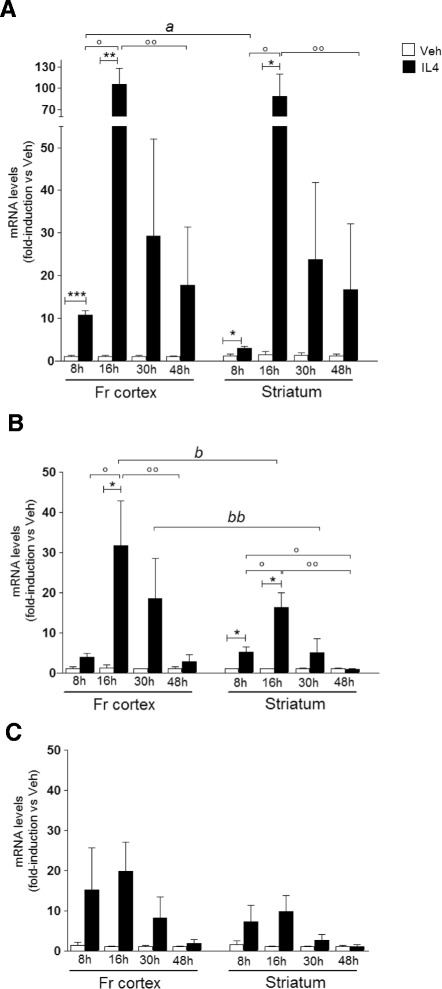
**Time course of IL4-induced M2 gene expression in frontal cortex and striatum**. Following the indicated time intervals after intracerebroventricular injection of saline (Veh) or IL4, the RNA extracted from the frontal cortex (Fr cortex) and striatum was analyzed by real time PCR to evaluate **(A)** *Fizz1*, **(B)** *Arg1*, and **(C)** *Ym1* gene expression. Data sets for each gene were calculated using the 2^-ddCt^ method with respect to the mean value of the 8 h vehicle group. Bars represent mean values ± SEM. * *P* < 0.05 versus Veh; ** *P* < 0.01; *** *P* < 0.0005 versus Veh; ° *P* < 0.05 versus IL4 8 h; °° *P* < 0.05 versus IL4 16 h (*n* = 4 to 6); *a P* < 0.0005 versus striatum 8 h; *b P* < 0.05 versus striatum 8 h; *bb P* < 0.05 versus striatum 16 h.

To evaluate whether microglia populating different brain regions are able to attain a similar M2 response, we analyzed M2 gene expression in the frontal cortex and striatum, in which M2 polarization was shown to occur under neurodegenerative conditions [23,24]. The mRNA levels coding for *Fizz1*, *Arg1*, and *Ym1* genes, known to be induced by IL4 in peripheral macrophages, were analyzed in a time course experiment following icv IL4 treatment. As shown in Figure 1A, *Fizz1* mRNA levels in the frontal cortex were significantly elevated following 8 h treatment and further increased after 16 h, while after more prolonged time intervals the effect of IL4, although not statistically significant, could still be observed and remained around 20-fold higher than vehicle-injected mice 48 h after the injection of IL4. Analogously, a significant induction of *Fizz1* mRNA levels was observed in the striatum at the earliest time points analyzed, with a temporal profile of mRNA induction that is superimposable to that observed in the cortex, except for the 8 h treatment; interestingly, at this time point the effect of IL4 is significantly different in the two brain areas, with the cortex resulting in a significantly higher gene induction than the striatum. The increased variability in *Fizz1* mRNA levels that is observed in both brain areas along with treatment duration could probably account for loss of statistical significance in the IL4 effect observed at 30 and 48 h after injection.

As shown in Figure 1B, the short-term treatments with IL4 resulted in a significant induction of *Arg1* mRNA levels in the brain; also, for this M2 gene, the IL4 activity is significantly more pronounced in the frontal cortex than in the striatum following 16 and 30 h treatment, although the response after 8 h treatment is statistically significant only in the striatum; unlike *Fizz1* induction, no difference between vehicle or IL4-treated mice was observed at the last time point analyzed. Analogously, the temporal profile of *Ym1* induction also supports diversity in the response to IL4 among the frontal cortex and striatum. In fact, *Ym1* mRNA levels are increased for up to 30 h in the frontal cortex, although never reaching statistical significance, while the effect of IL4 is weaker and faster in the striatum (Figure 1C). Thus, these data show that central administration of IL4 enables this signal to distribute from cerebral ventricles to distant brain regions, such as the frontal cortex, and allows evaluation of the induction profile of selected M2 genes and the estimation of differences in the M2 response in distinct brain areas.

**Figure 2:**
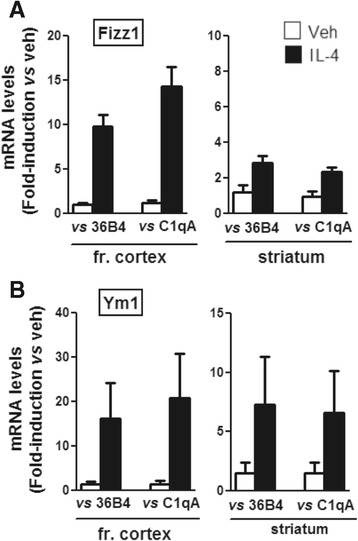
**Normalization of M2 gene expression using microglial gene** ***C1qA.*** At 8 h after intracerebroventricular injection of saline (Veh, open bars) or IL4 (IL4, black bars), RNAs from the frontal cortex and striatum were analyzed by Real Time PCR to evaluate Fizz1 **(A)** and Ym1 **(B)** gene expression. ΔCt values are calculated with respect to either the 36B4 housekeeping gene (versus 36B4) or C1qA microglia-specific gene (versus C1qA) and are shown as the 2^-ddCt^ method with respect to the mean value of the vehicle group. Bars represent mean values ± SEM (*n* = 4 to 6).

We next asked whether the different intensity of the IL4 response observed between striatum and frontal cortex could be ascribed to a difference in the number of microglia cells that reside in these areas. We calculated M2a gene expression in relation to the mRNA levels of *C1qA*, a gene that is highly expressed specifically in microglia [25] and that reflects the amount of microglia cells present in whole extracts of a given brain area; as shown in Figure 2, a similar potency and temporal profile of IL4 activity is observed in the striatum and frontal cortex to those shown in Figure 1, suggesting that the region-specific difference in IL4 responses cannot be ascribed to a different microglia cell number within the areas analyzed.

**Figure 3:**
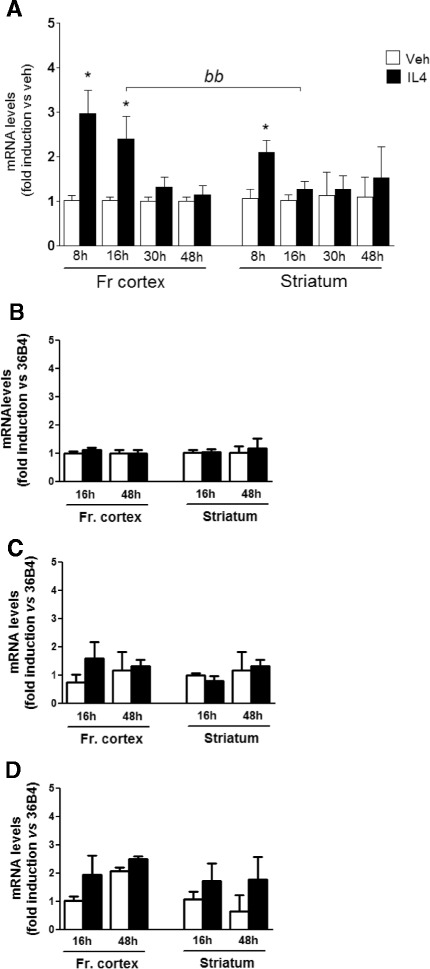
**Gene expression of M2a, M2c, and M1 polarization markers.** Expression of *CD206* **(A)**, *TGFβ* **(B)**, *IL1β* **(C)** and *TNFα* **(D)** genes was analyzed by real time PCR on RNA extracted from the frontal cortex (Fr cortex) and striatum of mice treated by intracerebroventricular injection for 16 and 48 h with saline (veh, open bars) or IL4 (black bars), as indicated. Data sets for each gene were calculated with the 2^-ddCt^ method with respect to the mean value of each vehicle group. Bars represent mean values ± SEM. * *P* < 0.05 versus veh 16 h; *bb P* < 0.05 versus striatum 16 h (*n* = 4 to 6).

Based on recent knowledge suggesting that microglia or macrophage activation exists as a spectrum of combinations of activation markers that are strictly dependent on experimental conditions [26], we evaluated the expression of additional genes, selectively involved in M2a, M2c, or M1 activation states [18]. As shown in Figure 3A, *CD206* (Mrc1) mRNA levels are significantly increased in the frontal cortex following 8 and 16 h treatment, while no effect is observed after longer treatments. This effect was expected based on the fact that *CD206* is also associated with the M2a phenotype induced by IL4; however, the induction level of this gene is much lower than those observed for the M2a genes, reported in Figure 1. Interestingly, *CD206* mRNA induction is observed in the striatum following 8 h, while IL4 activity is lost afterwards; this is consistent with a reduced IL4 response of this brain area as compared to the frontal cortex. The mRNA levels of TGFβ, as an M2c marker, as well as IL1β and TNFα, which are M1 response genes associated with inflammation, showed no alteration at any points of our time course experiment, as shown in Figure 3. In addition, we did not observe expression of IL10 mRNA in either vehicle- or IL4-treated mice, suggesting that this gene is not expressed in the brain and is not responsive to IL4 (data not shown). Thus, these data show the consistency of the icv IL4 experimental scenario with an M2a activation state, as that previously associated with IL4 activity in different macrophage populations; in addition, these results demonstrate that icv injections of vehicle or IL4 do not induce a classical inflammatory response, at least at the time points analyzed here.

Altogether, these data show that activation of the M2 response in the striatum is less efficient when compared with the frontal cortex in the experimental conditions utilized. Furthermore, our data show that the response to icv IL4 is gene-specific, suggesting that *Fizz1* is a more reliable and targetable marker of IL4-induced activation in the brain.

### Differences in IL4-induced M2 protein expression within brain regions and cells

**Figure 4:**
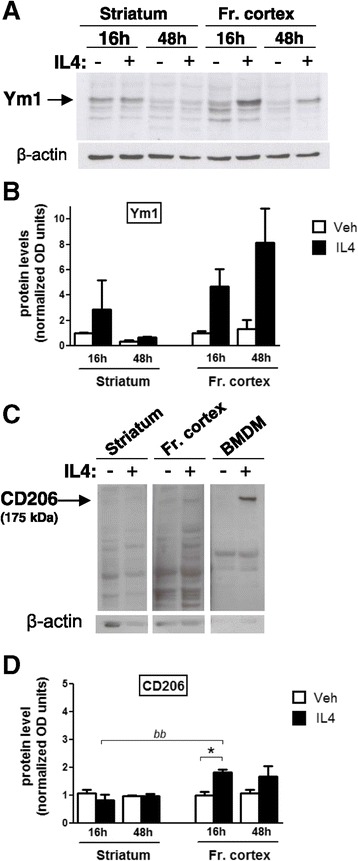
**Time course of IL4-induced M2a protein expression in mouse brain.** After intracerebroventricular injection of saline (Veh, open bars) or IL4 (black bars), the striatum and frontal cortex (Fr. cortex) were extracted at 16 and 48 h intervals and analyzed by Western blotting to evaluate Ym1 **(A**,**B)** and CD206 **(C**,**D)** protein levels. **(A**,**C)** Immunoblots using Ym1, CD206, and β-actin antibodies from representative samples are shown. The average densitometric value of the Ym1 **(B)** and CD206 **(D)** bands from Western blots of several samples was normalized to that of β-actin; bars represent mean values ± SEM; the arbitrary value of 1 was given to the value of the saline 16 h group for each protein and brain area analyzed (*n* = 4 to 6). BMDM, bone-marrow-derived macrophages.

We next analyzed whether IL4 activity in the brain correlated with an increase in M2 protein levels and whether the temporal profile of protein expression reconciled with that observed for the mRNA levels of M2 genes. To this aim, M2 protein levels were evaluated by Western blot analyses in the striatum and frontal cortex of mice injected with IL4; we were unable to assay Fizz1 and Arg1 proteins, owing to low specificity of the antibodies tested. As shown in Figure 4, a three-fold induction of Ym1 protein is observed in the striatum after 16 h, while Ym1 expression is similar to basal levels after 48 h treatment with IL4; on the contrary, 4.5 and 8-fold increases in Ym1 levels were observed in the frontal cortex after 16 and 48 h treatment, respectively, substantiating the diversity of the IL4 response in the two brain regions analyzed. In vehicle-treated mice, Ym1 protein levels (as well as mRNA levels; data not shown) were similar to those obtained in intact mice, with fold induction in protein expression values being 2.2 and 0.9 in the striatum and 0.8 and 0.9 in the frontal cortex at 16 and 48 h, respectively (data not shown), indicating that icv injection *per se* does not alter Ym1 expression, at least in the experimental condition assayed. The potency of the IL4 induction of Ym1 protein is in agreement with that related with Ym1 mRNA levels, which are shown in Figure 1C; in fact, similar induction levels of Ym1 protein are observed in the striatum and correlate with the lack of mRNA induction in this area following 30 and 48 h treatment (Figures 1C, 4B), whereas in the frontal cortex the persistence of Ym1 mRNA induction at 30 h correlates with the Ym1 protein increase assessed 48 h after treatment (Figures 1C, 4B). Thus, these results show that induction of Ym1 protein is more pronounced and persistent in the frontal cortex than in the striatum.

Importantly, the induction of CD206 protein by IL4 is also restricted to the frontal cortex, with no effect occurring in the striatum (see Figure 4C,D). The specificity of the CD206 protein signal is confirmed by loading protein extracts from vehicle- and IL4-treated bone-marrow-derived macrophage cells aside brain samples. Such a region-specific induction of CD206 to IL4 is consistent with the respective temporal profile of CD206 mRNA level induction in these areas (see Figure 3A). Thus, these data extend the assessment of the M2a activation profile induced by IL4 in brain and sustain the novel concept of the region-specific diversity in microglia polarization.

To evaluate whether the difference in the M2 response between the striatum and frontal cortex could be ascribed to an altered expression of IL4 receptor α (IL4Rα), mRNA levels of IL4Rα were evaluated in the striatum and frontal cortex of control and IL4-treated mice following 48 h treatment. Similar levels of IL4Rα mRNA were detected in these experimental groups (see Additional file 1), suggesting that the different M2 response to IL4 in the striatum and frontal cortex cannot be ascribed to a difference in IL4Rα expression.

Altogether, the difference in the Ym1 and CD206 levels between the striatum and frontal cortex demonstrates a region-specific difference in M2 polarization in response to IL4.

### IL4-induced M2 response is triggered by microglia cells

**Figure 5:**
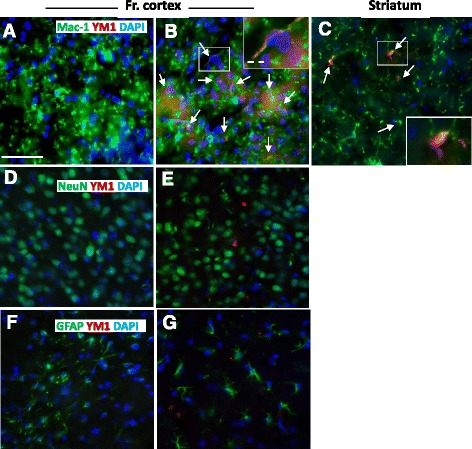
**Microglial localization and distribution of Ym1 protein in brain cells.** Brains after 16 h intracerebroventricular treatment with vehicle **(A**,**D**,**F)** and IL4 **(B**,**C**,**E**,**G)** were analyzed by co-immunostaining using Ym1 antibody **(A**-**G)**, here observed as red signals, together with cell specific antibodies Mac-1 **(A**-**C)**, NeuN **(D**-**E),** and GFAP **(F**,**G)**, revealed in green. Images were taken in the frontal cortex (Fr. cortex, **A**,**B**,**D**-**G)** or striatum **(C)**. The Ym1 signal specifically co-localized with Mac-1-positive cells, highlighted by white arrows and higher magnification inserts in B and C. Bar, 30 μm; dashed bar, 8 μm.

We then asked whether the observed induction of M2 gene expression by icv IL4 could be ascribed specifically to microglia. Double-labeling immunohistochemistry was used to localize Ym1 protein expression within specific brain cell types using antibodies against cell type-specific proteins, such as Mac-1, NeuN, and GFAP, known to be exclusively expressed by microglia, neurons, and astrocytes, respectively, each one was assayed together with an antibody against Ym1. As expected, the Ym1 signal was detected in microglial cells of the frontal cortex and striatum following 16 h of IL4 treatment, as shown in Figure 5A-C, with no evident morphological changes induced by IL4 in microglia cells. Conversely, we did not find any co-localization between Ym1 and neuronal or astrocytic markers (see Figure 5D-G). Interestingly, not all Mac-1-positive microglial cells display Ym1 expression following IL4 treatment; in fact, we estimated Ym1 responder cells being about 25 and 18% of the total number of Mac-1-positive microglia in the frontal cortex and striatum, respectively (data not shown) suggesting that a subpopulation of resident microglia corresponds to IL4-responding cells. Of notice, Ym1-positive microglia cells were preferably, although not exclusively, in close proximity to each other (see Figure 5B). Altogether, these results suggest that the brain M2 response to IL4 can be ascribed solely to a subset of microglia cells.

### IL4-induced M2 response is triggered by a subset of microglia cells

**Figure 6:**
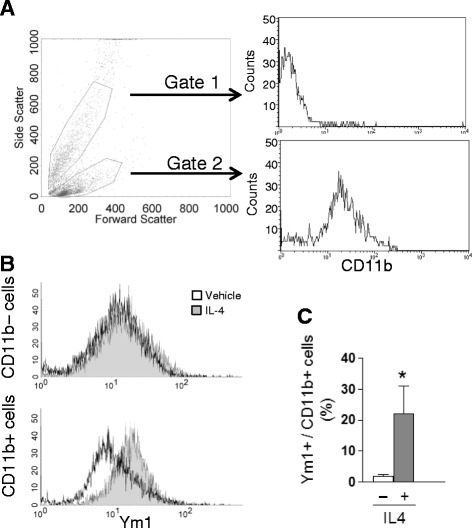
**Ym1 responder microglia subset.** Microglia cells were extracted from the cortices of mice treated for 16 h intracerebroventricularly with vehicle or IL4, purified by immunosorting using CD11b-loaded magnetic beads and analyzed by fluorescence-activated cell sorting for Ym1 protein expression. **(A)** CD11b-positive cells were characterized before purification and show a specific forward and side scatter profile with respect to other brain cells. **(B)** After immunosorting, IL4 treatment is ineffective in inducing Ym1 expression in the microglia-depleted fraction (upper panel). Conversely, IL4 induces an increase in Ym1 expression in CD11b-positive cells (lower panel). **(C)** Quantitative analysis of the number of Ym1-positive microglia cells shows that a significant percentage of cells (≈25%) is induced by the IL4 treatment. *: *P* < 0.05; *n* = 3.

To assess more precisely the percentage of IL4 responsive microglia cells, fluorescence-activated cell sorting analyses were performed on microglia purified from the cortex of adult mice 16 h after icv IL4 injection. Data show that the dissociated brain cells population before immunosorting contains CD11b-positive microglia that are about 12% of the total cell number and show a specific morphological and scattering profile, as expected (see Figure 6A). Magnetic beads loaded with anti-CD11b antibody allow sorting of a 90 to 95% pure microglia cell population (data not shown). Most importantly, staining with anti-Ym1 antibody on immunosorted microglia from saline or IL4 injected animals demonstrate that the Ym1 signal is only detected in a subset of microglia cells, as shown in Figure 6B. The average number of Ym1 responder cells after IL4 treatment is about 25% of the total microglia cells (see Figure 6C). Thus, these results further demonstrate the existence of a subpopulation of Ym1 responder microglia that undergoes polarization in response to IL4 *in vivo*.

**Figure 7:**
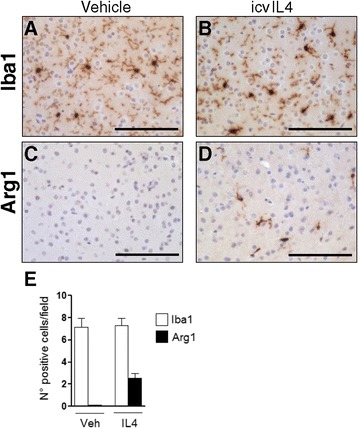
**Selective induction of Arg1 expression in a subset of microglia.** Brains after 16 h intracerebroventricular treatment with vehicle **(A**,**C)** and IL4 **(B**,**D)** were formalin fixed and embedded in paraffin and analyzed by immunohistochemistry to visualize Iba1 **(A**,**B)** or Arg1 **(C**,**D)** in the frontal cortex. Arg1 expression is observed selectively in microglia-like cells after IL4 treatment and is restricted to a subset of cells **(D)**. Scale bar 100 μm. The results obtained by counting the number of Arg1-positive microglia cells and the total number of Iba1-positive microglia cells demonstrated that an ≈ 35% subset of microglia cells is Arg1 responder microglia **(E)**. Veh., vehicle.

To extend our observation on the existence of subsets of IL4 responsive microglia, we analyzed the percentage of microglia expressing Arg1. As shown in Figure 7, immunohistochemical analysis using paraffin-embedded brain tissue enabled assessment that Arg1 expression is detected only after IL4 injection and solely in microglia cells, further confirming our results on the specificity of Ym1 expression in microglia. Most interestingly, by counting the number of Arg1-positive microglia cells and the total number of Iba1-positive microglia cells, we observed that a 35% subset of microglia cells is Arg1 responder microglia. In addition, these data also show that the percentage of IL4 responsive microglia may vary slightly, depending on the M2 marker analyzed.

Altogether these results demonstrate that the M2 polarization induced by IL4 in brain can be ascribed specifically to a subset microglia cells.

## Discussion

The aim of this study was to evaluate the extent to which microglia undergo M2 polarization *in vivo*. We thus developed a method that makes use of centrally administered IL4 to induce the M2a phenotype in brain. This experimental model allowed us to estimate the different ability of microglia populating the striatum and cerebral cortex to induce M2a gene expression and to identify the existence of subpopulations of microglia that support the IL4 response in brain.

Phenotype and functional plasticity is a characteristic of microglia; the presence of M1 and M2 phenotypes and a wide spectrum of intermediate and concomitant activation programs has been documented in animal models of cerebral diseases although with different ratios, temporal involvement and functional outcomes [23,24,27-30].

Activation of the brain M2 phenotype has been previously observed in animal models of neurological diseases and associated with suppression of inflammation, tissue remodeling and matrix deposition in a time- and environment-specific manner [23,24,27-30]. It is believed that M1 activation, that is, a protective innate immune response *per se*, might be exaggerated or unrestrained as a consequence of both acute CNS damage, such as after traumatic and ischemic reperfusion injuries, or under chronic neuroinflammation, as in Parkinson’s and Alzheimer’s diseases or multiple sclerosis; under these pathologic conditions several studies have shown that the anti-inflammatory and reparative response driven by alternative microglia polarization is dampened, as a consequence of the increased, self-propagating proinflammatory phenotype, revealing that the loss of an appropriate M2 response harms the dynamic and heterogeneous nature of microglia response and thus contributes to neurodegeneration [19]. However, the overall contribution of this response to neuroprotection and its specific involvement in preventing the development of neuropathological lesions within specific brain regions are still poorly understood.

Intriguingly, our data show that the M2a response in the striatum is less efficient than that observed in frontal cortex, in terms of M2a gene expression both at the mRNA and protein levels (see Figures 1, 2 and 4). One could thus hypothesize that microglia M2a response, being limited or inefficient within the nigrostriatal pathway, might be defective in the mechanisms that lead to reduction of neurotoxicity and to tissue repair and might thus contribute to the increased vulnerability of this neuronal population to neuroinflammation [2,20]. Indeed, neurotoxicity induced by the microglial M1 phenotype within the nigrostriatal pathway is further regulated by local cues, as demonstrated by dopaminergic neurons, which were shown to potentiate neurotoxicity under inflammatory pathologic conditions by oxidative dopamine metabolism. Dopaminergic neurons massively die in response to microglia M1 activation, whereas other neuronal populations involved in Parkinson’s disease etiopathogenesis survive the neuroinflammatory insult [8,16,31-33]. Our demonstration of a heterogeneous microglia M2a response lends further support to the hypothesis that region-specific responsiveness of microglia might be involved in the increased neuronal susceptibility to neuroinflammation.

Besides the nature, intensity, and persistency of the trigger, the responsiveness of microglia at a given time and within a specific anatomical location plays a central role in neuroinflammation. Activation of a specific phenotype is dictated by the number of responsive microglia and the ability of these responder cells to undergo polarization; it is believed that such properties are influenced by the specific environment in which microglia reside [2,9]. Adding complexity to this view, recent evidence supports the existence of subtypes of responder microglia within the same anatomical location, which are instructed by as yet undefined local signals to execute specific tasks that concur in housekeeping and inducible functions, such as immunological, clearing, and inflammatory actions [13-15]. In this scenario, our immunohistochemistry data provide a demonstration that the M2a response is specifically assigned to a subpopulation of microglia cells (see Figures 6 and 7). Although IL4 has been shown to induce astrocyte as well as neuron responses [34], our data suggest that the subset of microglia that triggers the M2a response represents a specific cellular target for improving regeneration and reducing proinflammatory neurotoxicity. Future investigations will therefore be relevant not only for understanding the physiology of these cells, but also for gaining insight into the biochemical pathways that govern the reactive potential of resident microglia subtypes. This information will allow the identification of novel therapeutic agents that increase neuron survival by targeting microglia reactivity [19] as well as the development of novel tracers that enable the functional imaging of the M2 phenotype in live animals [35].

In this perspective, icv IL4 administration represents a valuable experimental procedure to study the signaling pathways that control microglia M2 polarization. The use of this experimental model allows induction of the M2a response all over the brain, with no signs of inflammatory reactions in vehicle-treated subjects. Along with distribution, the rapidity, potency, and persistency of the M2a response observed following central administration of IL4 represent advantageous features in estimating the capability of microglia cells to acquire the M2a phenotype among and within different brain areas, in physiological as well as pathological conditions. Among the genes analyzed, *Fizz1* reached the highest induction levels in both the striatum and the frontal cortex. Expression of all M2a genes analyzed in this study has been shown to be dependent upon the IL4 signaling and coordinate action of IL4-inducible transcription factors [36-38]; yet, our data show that the IL4 signaling pathway that converges on *Fizz1* expression in the brain is highly efficient and, although its role in neuroinflammation is not clear [39,40], suggest that *Fizz1* may be considered as a reliable marker of IL4-induced M2a activation in brain.

## Conclusions

The results presented here show that central administration of IL4 induces a specific temporal pattern of M2a gene expression in striatum and frontal cortex, with *Fizz1* being the most inducible gene among those tested. Our results show that the responsiveness of brain macrophages to centrally administered IL4 might change among brain areas and that microglia M2a polarization can be ascribed to a subpopulation of IL4 responsive cells. Therefore, the biochemical pathways that instruct and enable microglia to undergo M2a activation represent key aspects in the physiopathology of microglia and challenging opportunities for the development of novel therapeutic and diagnostic agents.

## Additional file

Additional file 1:
**IL4Rα expression in the mouse brain.** The mRNA levels of IL4Rα were analyzed by real time PCR on the striatum and frontal cortex (fr. cortex) of control (Veh) and IL4-treated mice following 48 h treatment. Data are reported as ΔCt (dct) values with respect to the *36B4* housekeeping gene (*n* = 4).

## Abbreviations

ANOVA: analysis of variance
BSA: bovine serum albumin
CNS: central nervous system
DAB: 3,3′-diaminobenzidine
DAPI: 4′,6-diamidino-2-phenylindole
DMEM: Dulbecco’s modified Eagle’s medium
EDTA: ethylenediaminetetraacetic acid
FBS: fetal bovine serum
GFAP: glial fibrillary acidic protein
HBSS: Hank’s Balanced Salt Solution
HEPES: 4-(2-hydroxyethyl)-1-piperazineethanesulfonic acid
icv: intracerebroventricular
IgG: immunoglobulin G
IL1β: interleukin-1 β
IL4: interleukin-4
IL4Rα: IL4 receptor α
IL10: interleukin-10
IL13: interleukin-13
PBS: phosphate-buffered saline
PCR: polymerase chain reaction
PMSF: phenylmethanesulfonyl fluoride
SEM: standard error of the mean
TBS: Tris-buffered saline
TBST: TBS + 0.1% Tween 20
TGFβ: transforming growth factor β
TNFα: tumor necrosis factor α
