# Supplementary figures and images for: Heterogeneous induction of microglia M2a phenotype by central administration of interleukin-4
Source: J Neuroinflammation. 2014 Dec 31;11:211. doi: 10.1186/s12974-014-0211-6 (PMC4302702; doi:10.1186/s12974-014-0211-6)

## Slide 1
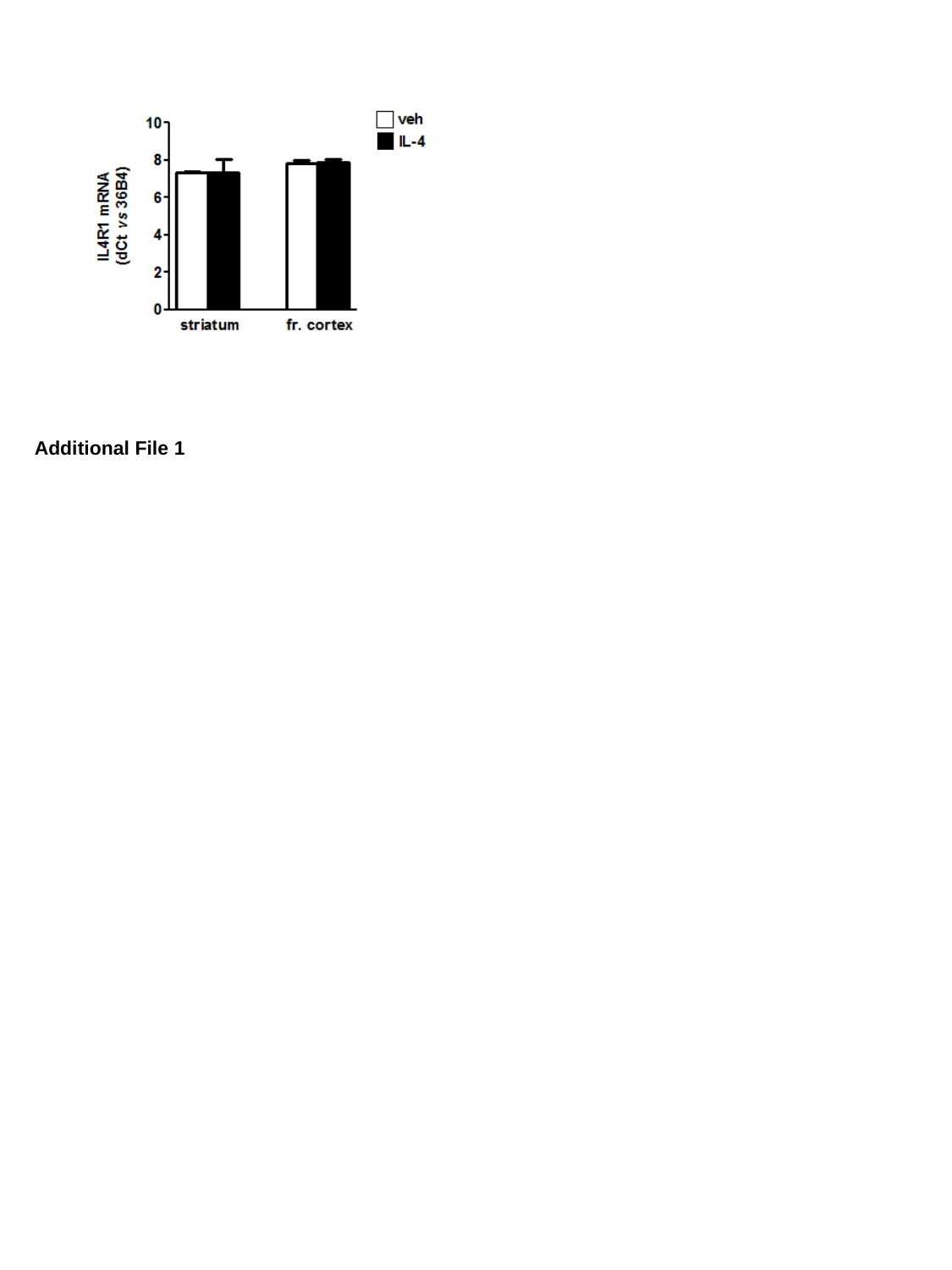

Additional File 1

Supplement: Additional file 1: — IL4Rα expression in the mouse brain. The mRNA levels of IL4Rα were analyzed by real time PCR on the striatum and frontal cortex (fr. cortex) of control (Veh) and IL4-treated mice following 48 h treatment. Data are reported as ΔCt (dct) values with respect to the 36B4 housekeeping gene (n = 4). [file 12974_2014_211_MOESM1_ESM.pptx]
